# Clinicopathological Features of Growth Hormone-Producing Pituitary Adenomas in 242 Acromegaly Patients: Classification according to Hormone Production and Cytokeratin Distribution

**DOI:** 10.1155/2013/723432

**Published:** 2013-01-21

**Authors:** Ryosuke Mori, Naoko Inoshita, Junko Takahashi-Fujigasaki, Tatsuhiro Joki, Hiroshi Nishioka, Toshiaki Abe, Takeshi Fujii, Shozo Yamada

**Affiliations:** ^1^Department of Neurosurgery, The Jikei University School of Medicine, 3-25-8 Nishishinbashi, Minato-ku, Tokyo 105-8461, Japan; ^2^Department of Pathology, Toranomon Hospital, 2-2-2 Tranomon, Minato-ku, Tokyo 105-8470, Japan; ^3^Division of Neuropathology, The Jikei University School of Medicine, 3-25-8 Nishishinbashi, Minato-ku, Tokyo 105-8461, Japan; ^4^Division of Hypothalamic and Pituitary Surgery, Toranomon Hospital, Tokyo 105-8470, Japan; ^5^Okinaka Memorial Institute for Medical Research, 2-2-2 Tranomon, Minato-ku, Tokyo 105-8470, Japan

## Abstract

The aim of this study was to clarify the relationship between the histological features of GH-producing adenomas surgically resected at the Toranomon Hospital and the clinical features of the patients. Histological examinations, including immunohistochemistry for anterior pituitary hormones and cytokeratin (CK), were performed on 242 consecutively excised GH-producing pituitary adenomas. Immunohistochemistry showed 45% of the adenomas to be monohormonal and 55% to be plurihormonal, producing GH-PRL (77%), GH-TSH (13%), and GH-PRL-TSH (10%). One-fourth of the monohormonal GH adenomas had a dot-like pattern of CK immunoreactivity in the majority of the tumor cells (>80%); they were significantly more common in female or younger patients and usually tended to be larger and more invasive than monohormonal GH adenomas with perinuclear CK. Interestingly, CK-immunonegative adenomas were found in only 5% of the patients; they also showed a tendency to be larger, suggesting that they are a distinct type of GH adenoma with clinically aggressive features. Serum hormone levels correlated well with tumor size only in GH-producing adenomas with a perinuclear pattern of CK immunoreactivity. Each histological subtype of adenoma, classified according to the pattern of CK immunoreactivity, was associated with distinct clinical characteristics. This information is useful for understanding the pathophysiology of acromegaly-causing GH-producing adenomas.

## 1. Introduction

Acromegaly is a syndrome caused by overproduction of growth hormone (GH), which is secreted, in majority of the patients, from GH-producing pituitary adenomas [1]. It is well known that GH-producing pituitary adenomas often coexpress prolactin (PRL) and, less frequently, thyroid stimulating hormone (TSH) [1]. Production of anterior pituitary hormones in nonneoplastic as well as neoplastic cells is controlled by several transcription factors and cofactors [2]. Accumulating evidence supports the hypothesis that normal and adenoma cells expressing GH, PRL, and TSH are regulated by the pituitary-specific transcription factor-1 (Pit-1) and, therefore, belong to the Pit-1 cell lineage [2–7].

Histologically, monohormonal GH-producing adenomas are classified in two subtypes, densely granulated (DG) and sparsely granulated (SG), based on the density of secretory granules in the cytoplasm of the adenoma cells [1, 8, 9]. These two types of tumors appear to have different clinical, endocrinological, and neuroimaging features [9–13]. It is therefore of importance to identify their histological subtypes. DG- and SG-type cells have different cytoskeletal features, with or without the formation of fibrous bodies, the hallmark of SG type cells, which are globular aggregations of intermediate filaments as seen on electron micrographs [13–17]. This spherical aggregate can also be detected as dot-like structure immunostained with an antibody against cytokeratin (CK). In DG type cells, CK immunostaining is perinuclear [12, 13]. Adenomas are diagnosed according to the ratio of DG- and SG-type features, which are conventionally distinguished by the pattern of distribution of CK [12, 13]. The distribution of CK also varies in plurihormonal GH adenomas. These adenomas are histologically subcategorized by electron microscopic analysis. However, the subtypes are difficult to predict on the basis of the CK pattern alone [8].

In this study, we examined pathological features of pituitary adenomas surgically resected from 242 patients with clinical symptoms of acromegaly. The adenomas were subcategorized according to hormone secretion and CK distribution assessed by immunostaining. Clinicopathological correlations with the clinical features of the patients were established retrospectively.

## 2. Materials and Methods

### 2.1. Patients and Clinical Data

Pituitary adenomas surgically removed from acromegaly patients admitted to the Toranomon Hospital (Tokyo, Japan) from 2008 to 2010 were analyzed. The patients' ages, sex, and hormonal profiles were obtained from clinical records. Basal plasma levels of GH, as well as insulin-like growth factor- (IGF-) 1 and PRL and TSH levels, were measured in the morning after overnight fasting. Maximum tumor diameter, tumor volume, Knosp grade, and sphenoid sinus invasion were evaluated. Sixty-five patients who had been treated before surgery (55 with somatostatin analogues, 8 with dopamine receptor subtype-2 agonist, 2 with both agents) were excluded from analysis of GH and IGF-1 levels, maximum tumor diameter, tumor volume, Knosp grade, and sphenoid sinus.

### 2.2. Imaging Studies

The maximum tumor diameter was measured by preoperative magnetic resonance imaging (MRI). The tumor volume was estimated on axial, coronal, and sagittal MRI sections as follows: 0.5 × width × length × height [18]. Cavernous sinus invasion by the tumor was classified by MRI according to Knosp et al. [19]. Sphenoid sinus invasion or bony destruction of the sellar floor was judged by a preoperative computed tomography (CT) scan. Patients with a documented history or MRI findings of pituitary apoplexy were excluded from this study.

### 2.3. Histological Studies

Surgically removed adenoma tissues were fixed in 10% formaldehyde and embedded in paraffin and cut in 3 *μ*m thick sections for hematoxylin-eosin (HE) and immunohistochemical staining. Immunohistochemistry was performed with the avidin-biotin-peroxidase method. Sections were incubated with the following antisera: anti-GH (Dako, Carpinteria, CA, USA; A0570), anti-PRL (Dako, Carpinteria, CA, USA; A0569), anti-beta subunit of TSH (Kyowa Medex Co., Ltd, Tokyo, Japan), anti-Adrenocorticotropic hormone (ACTH) (Dako, Carpinteria, CA, USA; A0571), anti-Follicle stimulating hormone (FSH) (BioGenex, San Ramon, CA, USA; MU026-UC), anti-Luteinizing hormone (LH) (Nichirei Biosciences Inc, Tokyo, Japan), anti-CK (CAM 5.2) (Becton Dickinson, San Jose, CA, USA), and anti-Pit-1 (BD Biosciences, San Jose, CA, USA). 

**Figure 1:**
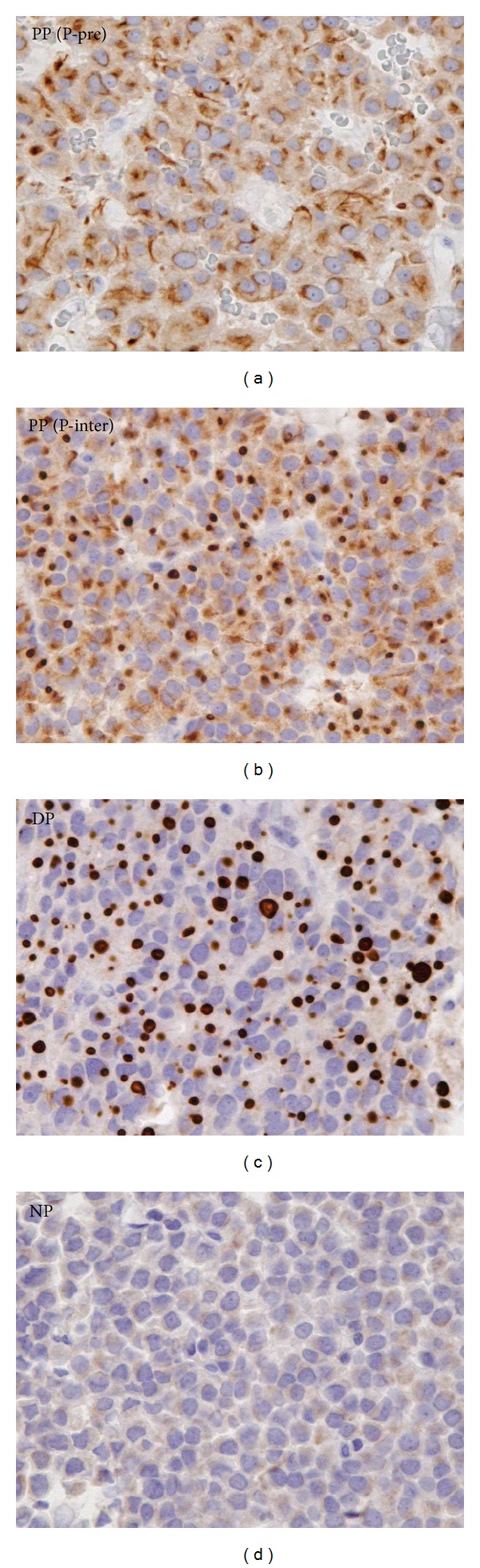
Immunohistochemical staining of cytokeratin (CK) in adenomas. In predominantly perinuclear (P-pre) perinuclear pattern (PP) adenomas most of the cells have perinuclear CK immunoreactivity. Intermediate (P-inter) PP adenomas contain a mixture of cells with either dot-like or perinuclear patterns of CK. In dot-like pattern (DP) adenomas, dot-like immunoreactivity was detected in more than 80% cells. In negative pattern (NP) adenomas, no significant CK immunoreactivity was detected.

Based on hormone staining, the adenomas were subclassified as GH, GH-PRL, GH-TSH or GH-PRL-TSH adenomas. Each group was further divided into 3 categories according to the pattern of CK staining: tumors with a perinuclear pattern of CK (PP), those with dot-like pattern of CK (DP), and those immunonegative for CK (NP). Adenomas are often composed of a mixture of cells with perinuclear and dot-like patterns; these have clinical features similar to adenomas with the predominantly perinuclear pattern cells, with regard to sex, age, hormone levels, tumor size, and invasiveness [20, 21], and were, therefore, included in the PP category. The latter was further subdivided into a perinuclear predominant group (P-pre), if more than 80% of the cells had perinuclear CK immunoreactivity, and a perinuclear intermediate group (P-inter), when 20% to 80% of the cells had a dot-like CK pattern. Adenomas in which more than 80% of the cells had a dot-like CK pattern were classified DP. The representative staining patterns of P-pre, P-inter, and DP and NP adenomas are shown in Figure 1. 

### 2.4. Statistical Analysis

The data were analyzed by the Chi square test, Mann-Whitney *U* test, and regression analyses. Differences were considered significant at *P* values <0.05.

## 3. Results and Discussion

### 3.1. Patient Profiles

The clinical data of the patients are summarized in Table 1. The age at diagnosis ranged from 18 to 77 years (mean ± SD: 48.0 ± 12.9), and the gender distribution was comparable (107 males and 135 females).

### 3.2. The Incidence of Monohormonal and Plurihormonal GH Adenomas

**Figure 2:**
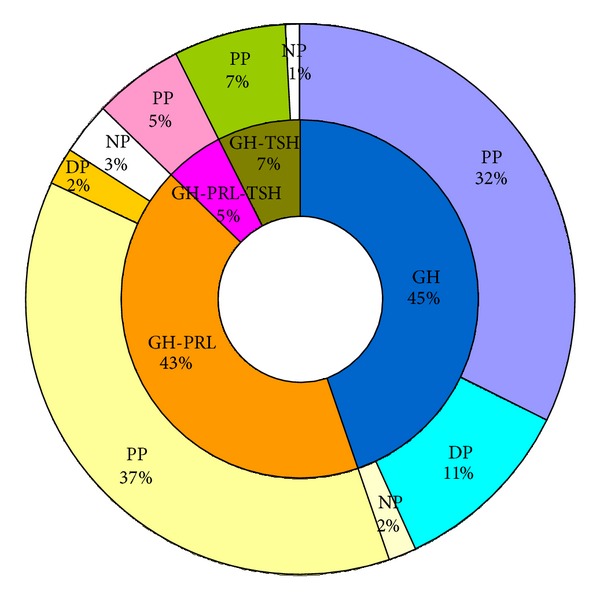
Frequency of adenoma subtypes categorized according to their hormone and cytokeratin staining patterns.

There were 108 monohormonal GH adenomas (45%) and 134 plurihormonal adenomas (55%) (Table 2 and Figure 2). The plurihormonal group contained three types of adenomas, GH-PRL (103/134 cases, 77%), GH-TSH (18/134 cases, 13%), and GH-PRL-TSH adenomas (13/134 cases, 10%). Plurihormonality is commonly observed in GH-producing adenomas, around half of which are reported to contain PRL [8]. Yamada et al. reported that 13 out of 31 growth hormone-producing adenomas (42%) were plurihormonal: GH-PRL (7/13 cases, 54%), GH-TSH (3/13 cases, 23%), and GH-PRL-TSH adenomas (3/13 cases, 23%) [13]. More than half of the GH-producing adenomas in our study expressed PRL and/or TSH. All of the examined adenomas were Pit-1-positive, indicating that the acromegaly-causing pituitary adenomas were of the Pit-1 lineage.

### 3.3. Categorization of the Adenomas by Their CK Patterns

The monohormonal and plurihormonal GH-producing adenomas were further subcategorized according to the distribution of CK immunoreactivity in the cytoplasm. In monohormonal GH adenomas, DG- and SG-type cells can be distinguished, respectively, by their perinuclear or dot-like patterns of CK immunoreactivity in the cytoplasm [10–14, 22–24]. 

We first examined difference between P-pre and P-inter profiles in the monohormonal and plurihormonal adenomas. There were no significant differences in age, sex, basal GH level, IGF-1 expression, maximum tumor diameter, tumor volume, Knosp grade (>3), and sphenoid sinus invasion, with the exception of IGF-1 levels in GH-PRL adenomas, which were higher in the P-pre than in the P-inter group (749 ± 297 versus 574 ± 203, *P* = 0.015). Thus, the P-pre and P-inter types had similar clinical features, justifying their inclusion in the category of PP adenomas. 

PP adenomas were the major subtype in all of the monohormonal and plurihormonal GH adenomas: 70% of GH, 89% of GH-PRL, 85% of GH-TSH, and all of GH-PRL-TSH adenomas. DP adenomas were less frequent: 25% of the monohormonal GH adenomas and 5% of GH-PRL adenomas. None of the GH-PRL-TSH and GH-TSH adenomas were classified DP. Only 5% (13/242) of the adenomas were negative for CK.

### 3.4. Clinicopathological Correlations

The results were summarized in Table 2. Monohormonal GH adenomas classed as DP had distinct clinical features. Although basal GH levels and IGF-1 levels did not differ significantly between PP and DP adenomas, the latter occurred frequently in females, at a significantly younger age, they were larger and more advanced according to the Knosp classification, consistent with previous reports [10–13, 22, 25]. One of the characteristic ultrastructural features of sparsely granulated adenomas is formation of fibrous body, which could be identified as a dot-like pattern by CK immunestaining. Thus, the DP GH adenomas represent sparsely granulated somatotroph adenomas known to demonstrate distinct clinical features. In contrast, the clinical features associated with PP and DP types of plurihormonal GH-PRL adenomas were similar. These results indicate that subclassification according to the distribution of CK will help to identify clinically aggressive monohormonal GH adenomas, as previously shown, but not plurihormonal adenomas. 

Among the CK-negative (NP) adenomas in our series, there were 4 GH, 7 GH-PRL, and 2 GH-TSH adenomas. The NP GH-PRL adenomas were significantly larger in size and had lower GH levels compared to PP GH-PRL adenomas. NP GH adenomas also tended to become larger than PP GH adenomas. NP GH-TSH adenomas did not differ significantly from PP GH-TSH tumors, but this might be attributed to the small number of such tumors in our series. Yoneda et al. reported a case of a Pit-1 lineage macroadenoma, with weak GH, PRL, and TSH immunoreactivity, and no cytoplasmic CK staining [26]; however, the clinicopathological features of NP GH-producing adenomas have not been well discussed. It will be necessary to accumulate more cases of CK-negative adenomas to determine whether the NP adenoma is a distinct type of pituitary adenoma with potentially aggressive clinical features.

### 3.5. Serum Hormonal Levels and Tumor Sizes

**Figure 3:**
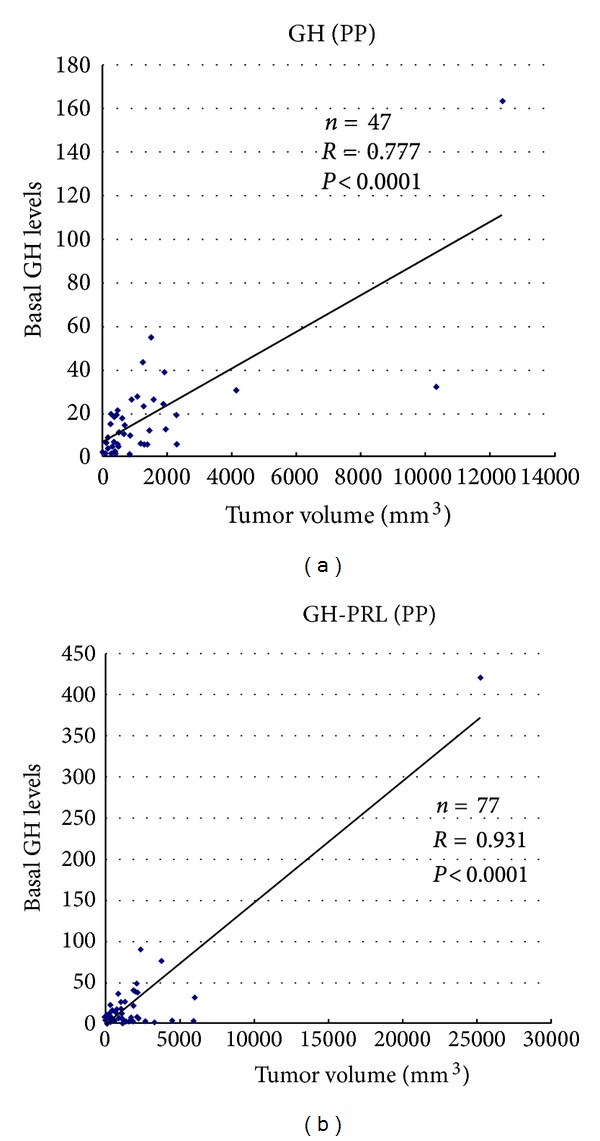
Correlation between basal GH levels and tumor volume in perinuclear pattern (PP) GH adenomas and GH-PRL adenomas.

**Figure 4:**
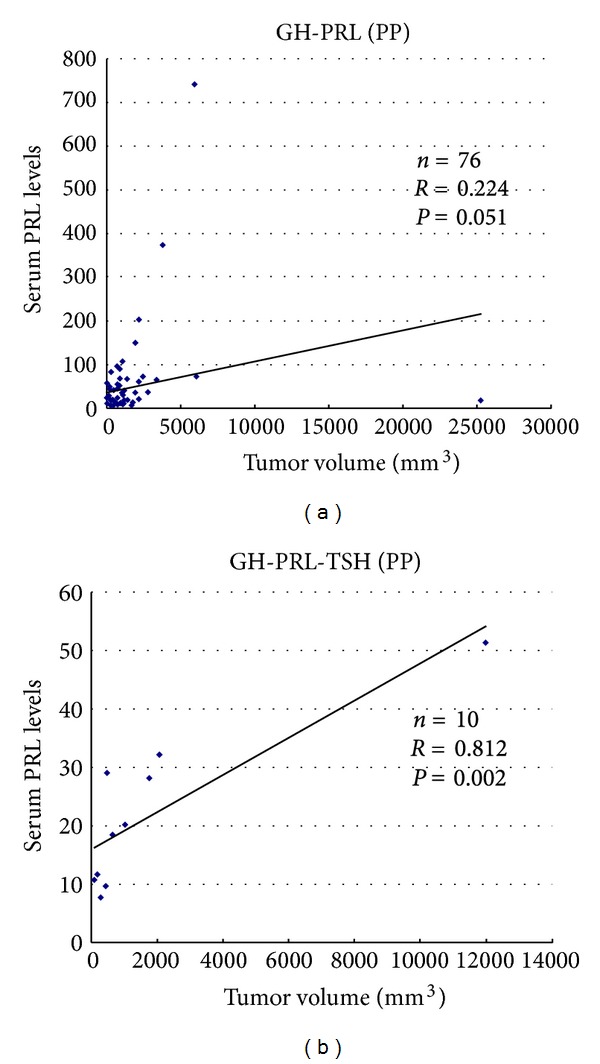
Correlation between serum PRL levels and tumor volume in perinuclear pattern (PP) GH-PRL and GH-PRL-TSH adenomas.

Unlike prolactinomas, clear correlations have not been established between the size of GH-producing adenomas and serum GH levels [9, 27, 28]. In our study, basal GH levels strongly correlated with tumor volume in PP type monohormonal GH adenomas and GH-PRL adenomas (*R* = 0.777  *P* < 0.0001 and *R* = 0.931  *P* < 0.0001, resp.) (Figure 3). In DP- and NP-type adenomas, however, correlation between basal GH levels and tumor volume was not observed. Serum PRL levels also correlated with tumor volume in PP-type GH-PRL-TSH adenomas and tended to correlate with tumor volume in PP GH-PRL adenomas (Figure 4). 

## 4. Conclusions

We examined the clinicopathological features of GH-producing adenomas resected from a large series of 242 acromegaly patients. All of the adenomas were of the Pit-1 lineage and consisted of monohormonal GH adenomas (45%) and plurihormonal (GH-PRL, GH-TSH, and GH-PRL-TSH) adenomas (55%). In the group of monohormonal GH adenomas, DP adenomas with CK-positive dot-like structures in a majority of the cells (>80%) were distinguished by the age and sex distribution of the patients, the size of the tumor, and its invasiveness. A small number of adenomas without CK immunoreactivity (5%) also showed a tendency to grow larger in size. Thus, cell typing according to the distribution of CK immunoreactivity will help to distinguish potentially aggressive GH-producing adenomas in acromegaly patients. 

## Figures and Tables

**Table 1:** Clinical data of all subjects.

Age	48.0 ± 12.9
Sex (M/F)	107/135
GH (basal) (ng/mL)	17.3 ± 48.3
IGF-1 (U/mL)	602 ± 259
PRL (ng/mL)	27.5 ± 63.1
TSH (*μ*IU/mL)	0.8 ± 0.7
Maximum tumor diameter (mm)	16.8 ± 8.6
Micro (−10 mm)/Macro (>10 mm)	62/180
Tumor volume (cm^3^)	2.2 ± 4.7
Knosp (3,4)	24%
Sphenoid sinus invasion	15%

M: male; F: female; GH: growth hormone; IGF-1: insulin-like growth factor-1; PRL: prolactin; TSH: thyroid stimulating hormone.

**Table 2:** Clinical, endocrinological, and histological summary of growth hormone producing adenomas.

Total cases (*n* = 242)																

Hormonal type	GH adenoma (*n* = 108)				GH-PRL adenoma (*n* = 103)				GH-TSH adenoma (*n* = 18)				GH-PRL-TSH adenoma (*n* = 13)			
Cytokeratin staining	PP (*n* = 78)				PP (*n* = 90)				PP (*n* = 16)				PP (*n* = 13)			
	P-pre (*n* = 46)	P-inter (*n* = 32)	DP (*n* = 26)	NP (*n* = 4)	P-pre (*n* = 26)	P-inter (*n* = 64)	DP (*n* = 6)	NP (*n* = 7)	P-pre (*n* = 7)	P-inter (*n* = 9)	DP (*n* = 0)	NP (*n* = 2)	P-pre (*n* = 5)	P-inter (*n* = 8)	DP (*n* = 0)	NP (*n* = 0)

Age	49.8 ± 13.0		41.3 ± 7.80^a^	46.2 ± 15.4	50.1 ± 13.1		45.8 ± 12.5	34.4 ± 15.2^b^	45.8 ± 12.0		—	41.5 ± 10.6	49.5 ± 13.1		—	—
	49.2 ± 12.5	50.7 ± 13.9			44.7 ± 14.6	52.2 ± 11.9			48.0 ± 12.6	44.1 ± 12.1			52.2 ± 7.25	47.8 ± 16.1		
Sex (M/F)	42/38		7/19^c^	3/1	40/50		2/4	2/5	8/8		—	1/1	4/9		—	—
	22/24	18/14			15/11	25/39			5/2	3/6			3/2	1/7		

Cases without preoperative medical therapy (*n* = 177)																

Hormonal type	GH adenoma (*n* = 67)				GH-PRL adenoma (*n* = 87)				GH-TSH adenoma (*n* = 13)				GH-PRL-TSH adenoma (*n* = 10)			
Cytokeratin staining	PP (*n* = 47)				PP (*n* = 77)				PP (*n* = 11)				PP (*n* = 10)			
	P-pre (*n* = 24)	P-inter (*n* = 23)	DP (*n* = 17)	NP (*n* = 3)	P-pre (*n* = 21)	P-inter (*n* = 56)	DP (*n* = 4)	NP (*n* = 6)	P-pre (*n* = 6)	P-inter (*n* = 5)	DP (*n* = 0)	NP (*n* = 2)	P-pre (*n* = 3)	P-inter (*n* = 7)	DP (*n* = 0)	NP (*n* = 0)

Age	54.4 ± 11.9		40.6 ± 7.69	51.6 ± 13.5	50.3 ± 13.3		45.5 ± 16.1	37.1 ± 14.7	46.3 ± 13.2		—	41.5 ± 10.6	47.3 ± 13.9		—	—
	54.4 ± 10.8	54.5 ± 13.2			45.7 ± 15.4	52.0 ± 12.1			48.0 ± 13.8	44.4 ± 13.8			50.0 ± 5.56	46.1 ± 16.5		
Sex (M/F)	25/22		5/12	3/0	35/42		1/3	2/4	5/6		—	1/1	3/7		—	—
	14/10	11/12			14/7	21/35			4/2	1/4			2/1	1/6		
GH (basal) (ng/mL)	17.6 ± 24.9		17.0 ± 14.7	25.0 ± 21.1	17.8 ± 48.8		14.6 ± 8.84	4.26 ± 3.00^d^	12.0 ± 10.9		—	2.65 ± 0.77	17.5 ± 14.0		—	—
	19.7 ± 32.0	15.3 ± 14.5			30.5 ± 89.6	13.1 ± 17.0			14.1 ± 12.9	9.44 ± 8.73			11.4 ± 9.23	20.1 ± 15.5		
IGF-1 (U/mL)	650 ± 269		631 ± 275	665 ± 332	626 ± 245		606 ± 144	497 ± 298	574 ± 264		—	479 ± 203	621 ± 187		—	—
	649 ± 251	652 ± 293			749 ± 297	581 ± 207			561 ± 299	589 ± 247			549 ± 82.6	653 ± 215		
PRL (ng/mL)	9.10 ± 3.87		15.4 ± 11.7	7.73 ± 2.10	46.0 ± 95.9^e^		55.4 ± 87.8	29.8 ± 45.1	44.2 ± 113		—	8.35 ± 4.17	21.9 ± 13.5^f^		—	—
	10.1 ± 4.63	8.13 ± 2.62			58.5 ± 161	41.6 ± 58.6			11.7 ± 8.60	83.1 ± 168			17.2 ± 13.1	23.9 ± 14.2		
TSH (*μ*IU/mL)	0.80 ± 0.87		0.57 ± 0.34	0.72 ± 0.16	0.90 ± 0.74		0.39 ± 0.28	0.63 ± 0.44	0.85 ± 0.49		—	0.78 ± 0.10	0.66 ± 0.25		—	—
	1.00 ± 1.12	0.54 ± 0.36			0.92 ± 0.62	0.89 ± 0.78			0.62 ± 0.42	1.11 ± 0.46			0.66 ± 0.41	0.66 ± 0.19		
Maximum tumor diameter (mm)	13.8 ± 6.06		21 ± 5.67^g^	19 ± 5.19^h^	13.8 ± 6.35		12.2 ± 2.06	17.1 ± 4.26^i^	17.9 ± 13.1		—	27.5 ± 27.5	14.7 ± 7.66		—	—
	15.2 ± 7.13	12.4 ± 4.47			15.6 ± 8.83	13.1 ± 5.07			23.3 ± 15.6	11.4 ± 5.50			11.3 ± 4.93	16.1 ± 8.47		
Micro (−10 mm)/ Macro (>10 mm)	17/30		0/17	0/3	33/44		1/3	0/6	3/8		—	1/1	4/6		—	—
	7/17	10/13			8/13	25/31			1/5	2/3			2/1	2/5		
Tumor volume (cm^3^)	1.28 ± 2.29		3.02 ± 3.43^j^	1.69 ± 1.11^k^	1.18 ± 3.00		0.51 ± 0.41	1.76 ± 1.56^l^	3.08 ± 5.20		—	1.09 ± 1.53	1.88 ± 3.60		—	—
	1.77 ± 3.09	0.78 ± 0.68			2.26 ± 5.44	0.78 ± 1.05			5.18 ± 6.49	0.56 ± 0.53			0.82 ± 1.05	2.33 ± 4.27		
Knosp (3,4)	13%		71%^m^	0%	17%		0%	33%	27%		—	50%	30%		—	—
	17%	9%			19%	16%			50%	0%			33%	29%		
Sphenoid sinus invasion	13%		12%	0%	18%		0%	0%	36%		—	50%	10%		—	—
	17%	9%			24%	16%			33%	40%			33%	0%		

GH: growth hormone; PRL: prolactin; TSH: thyroid stimulating hormone; PP: perinuclear pattern; DP: dot pattern; NP: negative pattern; P-pre: perinuclear predominant pattern; P-inter: perinuclear intermediate pattern; IGF-1: insulin-like growth factor-1; M: male; F: female.

^a^Significantly younger compared with PP GH (*P* = 0.002), GH-PRL (*P* = 0.0007), and GH-PRL-TSH (*P* = 0.01) adenomas. ^b^Significantly younger compared with PP GH (*P* = 0.01), GH-PRL (*P* = 0.01), and GH-PRL-TSH (*P* = 0.03) adenomas. ^c^Significant female predominance compared with PP GH (*P* = 0.03) adenomas. ^d^Significantly lower compared with PP GH (*P* = 0.01), GH-PRL (*P* = 0.03), and GH-PRL-TSH (*P* = 0.03) adenomas. ^e^Significantly higher compared with PP (*P* < 0.0001), DP (*P* = 0.01), NP (*P* = 0.01) GH adenomas, and PP GH-TSH (*P* = 0.02) adenomas. ^f^Significantly higher compared with PP (*P* = 0.0005), NP (*P* = 0.02) of GH adenomas, and PP GH-TSH (*P* = 0.04) adenomas. ^g^Significantly larger compared with PP GH (*P* < 0.0001), GH-PRL (*P* < 0.0001), GH-PRL-TSH (*P* = 0.004) adenomas, and DP GH-PRL (*P* = 0.004) adenomas. ^h^Significantly larger compared with PP (*P* = 0.04), DP (*P* = 0.01) GH-PRL adenomas. ^i^Significantly larger compared with PP of GH-PRL (*P* = 0.03) adenomas. ^j^Significantly larger compared with PP GH (*P* < 0.0001), GH-PRL (*P* < 0.0001), GH-PRL-TSH (*P* = 0.006) adenomas, and DP GH-PRL (*P* = 0.004) adenomas. ^k^Significantly larger compared with PP (*P* = 0.03) and DP (*P* = 0.03) of GH-PRL adenomas. ^l^Significantly larger compared with PP of GH-PRL (*P* = 0.04) adenomas. ^m^Significant cavernous sinus invasion compared with PP (*P* < 0.0001), NP (*P* = 0.02) GH adenomas, PP (*P* < 0.0001), DP (*P* = 0.01) GH-PRL adenomas, PP GH-TSH (*P* = 0.02) adenomas, and GH-PRL-TSH (*P* = 0.04) adenomas.
